# Cryopreservation does not change the performance and characteristics of allogenic mesenchymal stem cells highly over-expressing a cytoplasmic therapeutic transgene for cancer treatment

**DOI:** 10.1186/s13287-022-03198-z

**Published:** 2022-11-14

**Authors:** Yoon Khei Ho, Kin Man Loke, Jun Yung Woo, Yee Lin Lee, Heng-Phon Too

**Affiliations:** 1 Department of Biochemistry, National University of Singapore, Singapore, 117596 Singapore; 2 NUS Centre for Cancer Research (N2CR), Yong Loo Lin School of Medicine, National University of Singapore, Singapore, Singapore; 3 Gentle Oak Veterinary Clinic, Singapore, 270021 Singapore

**Keywords:** Cryopreservation, Engineered mesenchymal stem cells, Prodrug therapy

## Abstract

**Background:**

Mesenchymal stem cells (MSCs) driven gene directed enzyme prodrug therapy is a promising approach to deliver therapeutic agents to target heterogenous solid tumours. To democratize such a therapy, cryopreservation along with cold chain transportation is an essential part of the logistical process and supply chain. Previously, we have successfully engineered MSCs by a non-viral DNA transfection approach for prolonged and exceptionally high expression of the fused transgene cytosine deaminase, uracil phosphoribosyl transferase and green fluorescent protein (CD::UPRT::GFP). The aim of this study was to determine the effects of cryopreservation of MSCs engineered to highly overexpress this cytoplasmic therapeutic transgene.

**Methods:**

Modified MSCs were preserved in a commercially available, GMP-grade cryopreservative—CryoStor10 (CS10) for up to 11 months. Performance of frozen-modified MSCs was compared to freshly modified equivalents in vitro. Cancer killing potency was evaluated using four different cancer cell lines. Migratory potential was assessed using matrigel invasion assay and flow cytometric analysis for CXCR4 expression. Frozen-modified MSC was used to treat canine patients via intra-tumoral injections, or by intravenous infusion followed by a daily dose of 5-flucytosine (5FC).

**Results:**

We found that cryopreservation did not affect the transgene expression, cell viability, adhesion, phenotypic profile, and migration of gene modified canine adipose tissue derived MSCs. In the presence of 5FC, the thawed and freshly modified MSCs showed comparable cytotoxicity towards one canine and three human cancer cell lines in vitro. These cryopreserved cells were stored for about a year and then used to treat *no-option-left* canine patients with two different types of cancers and notably, the patients showed progression-free interval of more than 20 months, evidence of the effectiveness in treating spontaneously occurring cancers.

**Conclusion:**

This study supports the use of cryopreserved, *off-the-shelf* transiently transfected MSCs for cancer treatment.

**Supplementary Information:**

The online version contains supplementary material available at 10.1186/s13287-022-03198-z.

## Background

Mesenchymal Stem Cells (MSCs) have long been reported to be hypo-immunogenic and show excellent tumour tropism. Despite their attractive tumour trophic and anti-angiogenic properties [1], the use of unmodified MSCs to treat cancer is highly debatable where there are conflicting reports of MSCs suppressing [2, 3] and enhancing [4, 5] growth of tumors. Increasingly, genetically modified MSCs have been shown to provide safer and more potent cancer therapy than the heterogenous native MSCs [6–8]. Particularly, stem cell driven GDEPT has advanced to phase II trial for the treatments of gastrointestinal cancer [9] and glioblastoma [10]. In these studies, stem cells were stably transduced to deliver suicide genes to the tumour sites for local conversion of non-toxic prodrug to anti-cancer agents.

Previously we developed a highly efficient cationic polymer-based method for the non-viral engineering of MSCs using DNA encoding CD::UPRT::GFP for safer foot-printless GDEPT. The freshly modified MSCs demonstrated high anti-cancer efficacy in both in vitro and in vivo studies [11, 12]. The payload of these transiently modified MSCs was significantly much higher than lentiviral modified cells [12]. Our next goal was to determine the feasibility of cryopreservation of these transiently modified MSCs for storage and ease of distribution. The successful demonstration of cryostorage of non-virally modified MSCs expressing CD::UPRT::GFP would be a stepping stone towards industrialization of an *off-the-shelf* product. Development of cell and gene therapy products that are readily available is predictably desirable as it ensures treatment accessibility where the products are manufactured at large scale, cryopreserved and readily distributed. From a commercial perspective, biobanking of cell products facilitates cell distribution and ensures immediate, steady *off-the-shelf* supply [13, 14]. From a regulatory perspective, cryopreservation offers assurance on the safety of the therapeutic doses by providing time for product standardisation, in vitro assays validation and quality control testing [14, 15].

Generally, the conventional practice of cryostoring in dimethyl sulfoxide (DMSO) has been shown to preserve the viability and differentiation potential of unmodified MSCs [16, 17]. Additionally, viral modified MSCs stably expressing a transgene by chemical selection has been successfully cryopreserved without affecting their transgene expression, cell viability and tumour tropism ability of MSCs [18]. Nonetheless, the impact of cryopreservation on transiently modified, high transgene expression MSCs may differ from their viral modified equivalents. Unlike stably modified MSCs, transiently modified MSCs are required to be cryopreserved post transfection, without further antibiotic selection or expansion. As there is no genome integration, transgene expression can be reduced or lost post-cryopreservation [19]. Additionally, transfection reagents may compromise the integrity of cell membrane, raising concerns over the possibility of reduced cell viability during cryopreservation [20, 21]. Metabolic burden imposed by significant exogenously expressed gene load can have detrimental effects [22], rendering the cells to be more vulnerable to the freezing and thawing process.

To date, the effect of cryopreservation on transient, highly over-expressed gene modified MSCs has yet to be clearly defined. A recent study reported that cell viability and transgene expression of the thawed cells are transfection protocol dependent [23]. Thus, it is crucial to assess the impact of cryopreservation in accordance with the target product profile. To explore the effects of cryopreservation on the highly over-expressed MSCs for GDEPT, we assessed cell viability, transgene expression, anti-cancer potency and tumour trophic functions of thawed non-virally modified canine MSCs. We used canine MSCs as we provide treatments to “no-option-left” canine patients with naturally occurring tumours.

## Methods

### Cell culture

Canine adipose tissue-derived mesenchymal stem cells (MSC) were purchased from Cellider Biotech S.L. and cultured in MEM alpha (BioWest) supplemented with 20% FBS (Hyclone) [24]. Commercial cell lines used for co-culture are A549 (human lung adenocarcinoma; ATCC CCL-185), Hs 888.T (human osteosarcoma; ATCC CRL-7622), RPMI 2650 (human nasal septum squamous cell carcinoma; ATCC CCL-30) and CLAC (canine lung adenocarcinoma; JCRB 1453). Except for RPMI 2650 that was maintained in EMEM (Lonza) supplemented with 10% FBS, all other commercial cell lines were cultured in DMEM (BioWest) supplemented with 10% FBS. Cells were maintained at 37 °C in humidified atmosphere and 5% CO_2_.

### Transfection procedure

Passage 4 of MSCs were transfected with CD::UPRT::GFP plasmid as previously described [11]. A total of 5.5 × 10^6^ cells were seeded in a 500 cm^2^ dish (Corning 431,111) 24 h prior to transfection. Polyethylenimine MAX (PEI, Polyscience) was added to serum free MEM alpha at 4µL of PEI (1 mg/mL) to 1 µg of plasmid DNA. A total volume of 5 mL of the mixture was incubated at room temperature for 15 min. After incubation, the transfection mixture was supplemented with transfection Enhancer. The Enhancer consist of DOPE/CHEMS (9:2 molar ratio Fusogenic lipid, Polar Avanti Lipid) and 1 µM Bufexamac (Histone deacetylase inhibitor; HDACi, Sigma-Aldrich) [25]. Cells were incubated for 24 h before analysis.

### Cryopreservation of MSC overexpressing CD::UPRT::GFP

Cells were washed twice with Plasma-Lyte A (Baxter 2B2544X) and harvested using TrypLE Express. After centrifugation, the cell pellets were resuspended in cryopreservation media, CS10 (CryoStor10), at a concentration of 1 × 10^6^ cells/mL, 2 × 10^6^ cells/mL or 3 × 10^6^ cells/mL in cryovials. The cryovials were transferred into a Mr. Frosty™ Freezing Container (Thermo Fisher Scientific) for overnight freezing in − 80 °C, before being stored in liquid nitrogen vapour for at least 5 months and up to 1 year. Before use, the cells were thawed using the ThawSTAR Automated Thawing System (Biolife Solutions), resuspended in 4 mL of Plasma-Lyte A, followed by centrifugation at 300 × g for 5 min. The pelleted cells were either resuspended in HypoThermosol Preservation Solution (Sigma-Aldrich) for cell viability assessment, administration for canine patients or suspended in culture medium for functionality analyses.

### Expression analysis

#### Flow cytometry

Cells were washed twice with Plasma-Lyte A (Baxter 2B2544X) and harvested using TrypLE Express. Single-cell suspension was obtained by passing through a 100 µm cell strainer (SPL). Percentage of fluorescent positive cells was quantified by CytoFLEX LX flow cytometer (Beckman Coulter) and the raw data was analyzed using non-modified MSCs as negative controls at < 0.9%, using Attune™ NxT Software (Version 3.1.2 Thermo Fisher Scientific). At least 10,000 cells were analyzed per sample.

#### Imaging

Cell images were taken with EVOS FL Cell Imaging System (Thermo Fisher Scientific) equipped with fluorescent light cubes for viewing of DAPI (Ex357/Em447) and GFP (Ex470/Em510) fluorescence.

### Cell viability assessment

To examine the stability of post-thaw modified cells, cryopreserved modified cells were thawed, centrifuged, and left in suspension at room temperature or 4 °C for up to 4 h. The cells were then stained with acridine orange (AO; 500 µM/mL; Sigma-Aldrich) and 4′,6 diamidino-2-phenylindole (DAPI; 250 µg/mL; Thermo Fisher Scientific) and quantified using the NucleoCounter® NC-3000 Automated Cell Counter. The viability was assessed using NucleoView (ChemoMetec).

### Phenotypic characterization

To determine the effect of cryopreservation on MSC phenotype, unmodified, freshly modified, and cryopreserved modified cells were fixed in 4% formaldehyde (Sigma-Aldrich) and stained with antibodies. Suspension cells were centrifuged at 800 × g for 5 min; and resuspended in 1 mL of Blocking Buffer containing 15% FBS in 1XPBS (BioWest). Cells were kept on ice for 15 min; washed with Flow Wash Buffer consisting of 1% FBS in 1XPBS. The cell pellets were then resuspended at a concentration of 1 × 10^6^ cells/mL in Flow Wash Buffer after removing the supernatant. Cells were either directly or indirectly stained with antibodies that recognize the specific cell surface markers. For direct staining, cells were stained with isotypic control (PE, Thermo Fisher Scientific), CD90 (PE, Thermo Fisher Scientific, clone YKIX337.217) and CD34 (PE, BD Biosciences, clone 1H6). For indirect staining, cells were stained with primary antibody of CD44 (Bio-Rad, clone YKIX337.8.7) and secondary antibody of PE (Thermo Fisher Scientific). All samples were washed, then resuspended in Plasma-Lyte A, and stored at 4 °C prior to analysis by flow cytometry.

### Trilineage differentiation assay

Cryopreserved cells were thawed, washed with cell culture media, centrifuged, and plated in media at 37 °C in humidified atmosphere and 5% CO^2^.

#### Adipogenic differentiation

Cells were plated at 1 × 10^5^ cells/well in a 24-well plate in culture media for 24 h. Media was removed and replaced with culture media (control wells) or StemPro™ Adipogenesis Differentiation Kit (Thermo Fisher Scientific). Media for each sample was changed every 3 days and cells were maintained for 14 days. Oil droplet was stained with Oil Red O solution and assessed by microscopy.

#### Osteogenic differentiation

Cells were plated at 1 × 10^5^ cells/well in a 24-well plate in culture media and permitted to adhere overnight before differentiation induction. Media was removed and replaced with culture media (control wells) or StemPro™ Osteogenesis Differentiation Kit (Thermo Fisher Scientific). Media was changed every 3 days. Calcium deposits were stained by Alizarin red S solution at day 21 post induction.

#### Chondrogenic differentiation

A total of 1 × 10^5^ cells/well was seeded in an ultra-low attachment round bottom 96-well plate in culture media 24 h prior to induction. Media was removed and replaced with culture media (control wells) or StemPro™ Chondrogenesis Differentiation Kit (Thermo Fisher Scientific). Media was changed every 3 days and cells were maintained for 21 days. The formation of cartilage, aggrecan, was stained using copper-containing Alcian Blue dye.

### Cell migration assay

MSC overexpressing CD::UPRT::GFP tumour tropism was assessed using 8.0 µm Transwell (Corning). A total of 4 × 10^5^ cells/well of cancer cells, A549 and RPMI 2650, were seeded in complete media in the lower chamber of the 24-well plate. After 24 h of incubation, the cultures were washed twice with 1XPBS and replaced with serum free DMEM. Unmodified, freshly modified and/or cryopreserved MSC overexpressing CD::UPRT::GFP, were seeded at 1.5 × 10^5^ cells/well into the upper chambers in serum free media. Cells that migrated to the bottom of the upper chamber were fixed with 4% PFA and stained with 10 µg/mL Hoechst 33,342 (Thermo Fisher Scientific). Three images per well were taken with EVOS FL Cell Imaging System (ThermoFisher Scientific) at 10 × magnification and the migrated cells were counted using ImageJ.

### In vitro* anticancer efficacy of cryopreserved CD::UPRT::GFP producing MSCs*

The anticancer efficacy of MSC overexpressing CD::UPRT::GFP in vitro was determined by direct co-culture with 4 cancer cell lines (A549, Hs 888.T, RPMI 2650 and CLAC). Here, 2 × 10^3^ cells/well of A549, Hs 888.T and CLAC, and 4 × 10^3^ cells/well in RPMI 2650 were seeded in 96-well plate 5 h prior to the seeding of fresh or cryopreserved MSC overexpressing CD::UPRT::GFP at the ratios of 1 MSC to 1, 5, 10, 50 and 100 cancer cells. After 24 h, the culture media was replaced with DMEM supplemented with 10% FBS, with or without 5-FC (150 µg/mL). The anticancer efficacy was measured by MTS assay after 6 days of co-culture.

### Use of GDEPT in canine patients

All the pet owners signed an informed consent authorizing treatment and were informed of the possible risks and side effects, and potential complications of the procedure. The body weight and tumor size of the canine patient were measured before and after the treatment. The x-rays or other diagnostic images of the pet patients were collected before, during and after the course of the treatment. The pet patients were given 500 mg per day of non-toxic prodrug 5FC orally for four consecutive days. The treatment was completed in 3 cycles. The health status, including daily activity and appetite of the treated pets was recorded by the owners. Follow-up of the patients’ well-being for at least 1 year included owners’ subjective observations and diagnoses from the veterinarian.

#### Intratumoural injection

For intratumoural injection, cryopreserved modified cells were thawed, centrifuged, and resuspended in 0.5 mL of HypoThermosol Preservation Solution in an insulin syringe. The cryopreserved MSC overexpressing CD::UPRT::GFP (1 × 10^7^ cells) were injected intratumourally at several spots around tumour mass.

#### Intravenous injection

For intravenous injection, cryopreserved modified cells were thawed, centrifuged, and resuspended in 7.5 mL of HypoThermosol Preservation Solution. The cryopreserved MSC overexpressing CD::UPRT::GFP (1.5 × 10^7^ cells) were injected intravenously through a cephalic vein. The cephalic vein was cleaned with an alcohol swab for sterilization and better visualization. A 27 gauge was inserted into the vein valve stylet and was secured by pasting micropore tape on the catheter. The other end of the extension tube was then attached to the catheter. The whole extension tube was inserted into a syringe pump system of which the flow rate can be controlled. The rate of infusion was set at 3 mL/h. A washing step of 2 mL saline was implemented after the syringe was empty to ensure the remaining volume of modified MSCs in the extension tube to be infused into the vein. The canine patient was monitored for 1 h for potential side effects.

### Statistical analysis

An unpaired two-tailed Student’s t-test was used, with the assumption that changes in the readout are normally distributed. All the graphs, calculations, and statistical analyses were performed using GraphPad Prism software version 9.4.1 for Windows (GraphPad Software, San Diego, CA, USA). All experiments were repeated at least thrice to determine reproducibility and to rule out random error.

## Results

### Modification and cryopreservation of canine MSCs

**Fig. 1:**
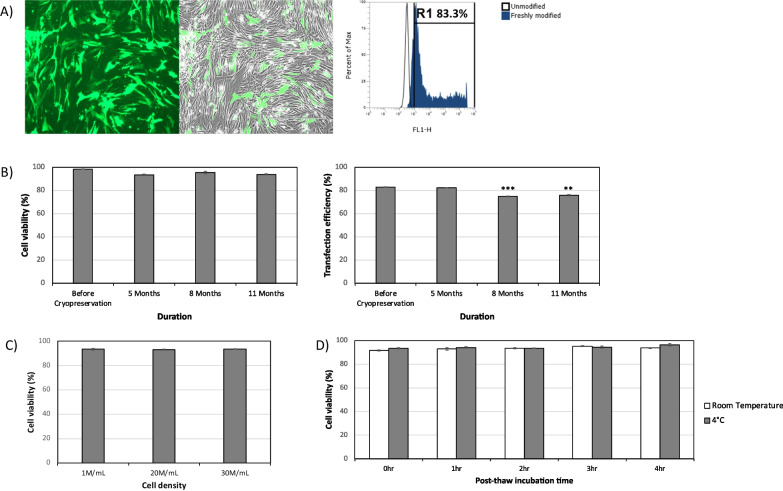
Cryopreservation and storage stability of MSC overexpressing CD::UPRT::GFP. (**A**) Cell images and transfection efficiency FACS profile of MSC overexpressing CD::UPRT::GFP before cryopreservation. Briefly, cells were plated and left overnight before addition of polyplex. 24 h post transfection, cells from multiple dishes were harvested and combined to obtain sufficient cells for storage and analysis. Flow cytometry (FACS) analysis was performed using CytoFLEX LX equipped with a FITC channel for GFP detection. Representative images are displayed. (**B**) Cell viability percentage was assessed using dye-exclusion assay (AO/DAPI, Chemometec) and transfection efficiency (flow cytometry analysis) for cryopreserved MSC overexpressing CD::UPRT::GFP at 5 months, 8 months and 11 months post cryopreservation. Significant differences in transfection efficiencies between modified MSCs before and post cryopreservation were calculated using two-tailed Student’s t test. **P < 0.005, ***P < 0.001. (**C**) MSC overexpressing CD::UPRT::GFP was stocked at 1 M, 20 M and 30 M/mL, and cell viability was measured 5 months post cryopreservation. (**D**) Post-thaw modified cells were spun down to remove CPA and resuspended in hypothermic storage solution HTS-FRS to assess stability at room temperature and 4 °C. Data of biological triplicates (*n* = 3) were expressed as mean ± SD

Canine adipose tissue derived MSCs were engineered to express CD::UPRT::GFP using a cationic polymer-based method as described previously [11]. When MSCs were modified with CD::UPRT::GFP plasmid instead of mRNA, the cells were found to express significantly higher payload (Additional file 1: Fig. S1). In view of the therapeutic benefit of having high payload in the MSCs, CD::UPRT::GFP plasmid was used in the current work. Two days post transfection, cells were harvested and cryopreserved in CryoStor10 (CS10), a commercially available GMP grade cryopreservative solution containing 10% DMSO. CS10 has previously been shown to maintain high post-thaw viability in multiple cell types [26–28]. Additionally, multiple clinical applications reported storage of cell product in CS10 [29, 30], making it an attractive reagent to be explored in our study. The transfection efficiency and cell viability after trypsinization were > 80% and > 95%, respectively (Fig. 1A). While the transfection efficiency reduced slightly to 75%, the viability of frozen-thawed modified cells was maintained at > 90% for more than 11 months post cryo-stored in liquid nitrogen (Fig. 1B).

In view of the need to prepare engineered MSCs at various dosages [18], cryopreserved MSC overexpressing CD::UPRT::GFP at varying densities, 1 × 10^6^, 2 × 10^7^, 3 × 10^7^ cells per mL per vial showed comparable post-thaw viabilities of > 90% (Fig. 1C). To determine durability of cryopreserved cells in suspension, we resuspended the cells in HypoThermosol® FRS (HTS) and found that the cell viability was maintained > 90% for up to 4 h at room temperature or at 4 °C (Fig. 1D). HTS consists of a proprietary ionic mixture, non-permeable substances, pH buffers, metabolites such as adenosine and glutathione to enable survival of various cell types during hypothermic storage at 2–8 °C [31]. It has been shown that HTS is safe for low volume intravenous injection [32], making it a potential biopreservative for cell product formulation.

Collectively, these results indicated that the modified cells can be stored at clinically relevant quantities and remained stable post-thaw for up to 4 h, providing ample time for processing and preparation prior to administration.

### Cryopreserved MSC overexpressing CD::UPRT::GFP retains MSC markers and differentiation potential

**Fig. 2:**
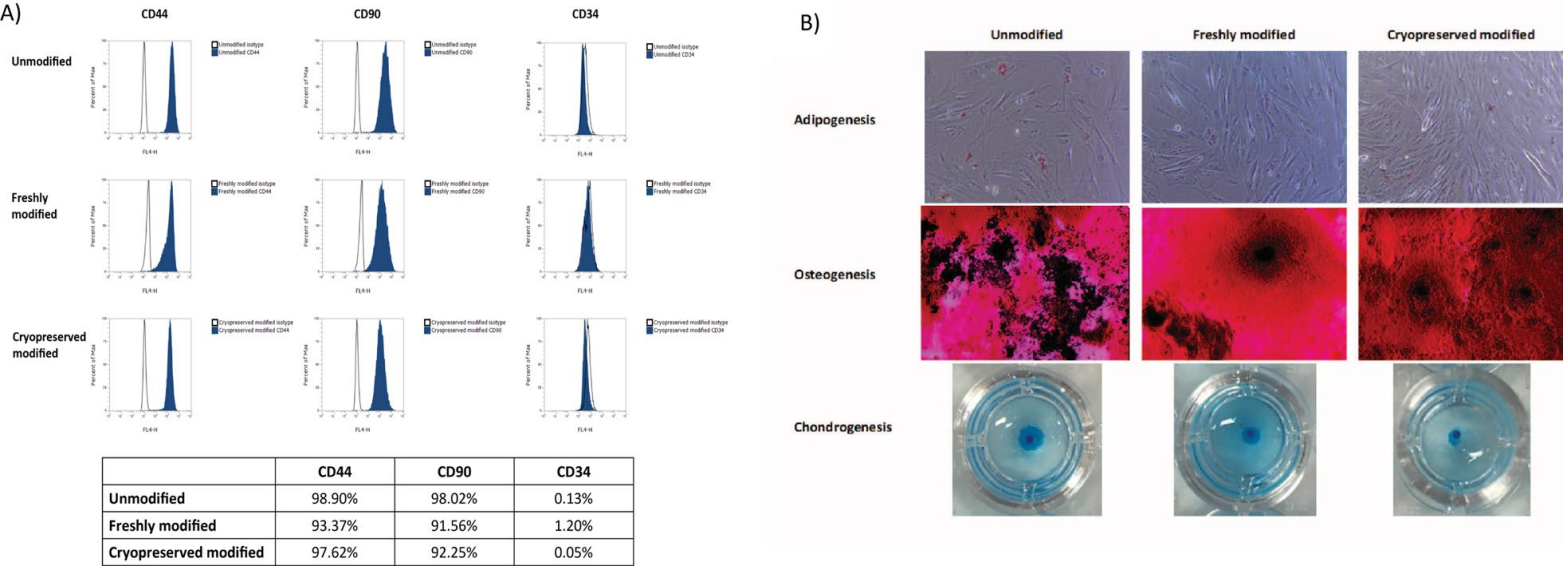
Cryopreservation of MSC overexpressing CD::UPRT::GFP does not alter MSC markers and differentiation potential. (**A**) MSC positive markers CD44 and CD90, and negative marker CD34 were stained using PE-conjugated antibodies and assessed using flow cytometric analysis against an isotype control. Table indicates percentage of CD44 + , CD90 + and CD34 − cells within the respective samples. (**B**) Trilineage differentiation potential of cryopreserved MSC overexpressing CD::UPRT::GFP relative to unmodified and freshly modified samples. Oil Red O (Sigma), Alizarin Red S (Sigma) and Alcian Blue (Sigma), were used to stain for oil droplets, calcium deposits and proteoglycan in each of the differentiated samples, respectively. Experiment was performed in triplicates (*n* = 3), representative images are presented

To ensure that modification and cryopreservation did not alter the cellular properties of the MSCs, we assessed the cell surface markers and differentiation potential of thawed MSC overexpressing CD::UPRT::GFP. In line with a report by Screven and colleagues [33], we found CD44 and CD90 to be > 90% expressed in all samples whereas, CD34 was expressed at < 2% (Fig. 2A). It is apparent that the expression of these markers on cryopreserved MSC overexpressing CD::UPRT::GFP did not vary from the freshly modified or unmodified samples. Trilineage differentiation of freshly modified and frozen modified MSCs also did not vary significantly from native MSCs (Fig. 2B). These results demonstrated that neither modification nor cryopreservation altered the phenotypic properties of the modified cells significantly.

### Cryopreservation does not affect cell recovery and therapeutic protein expression of the MSC overexpressing CD::UPRT::GFP

**Fig. 3:**
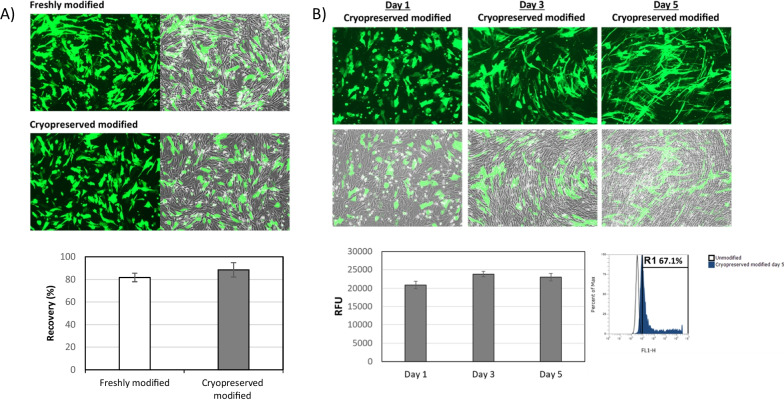
Cryopreserved MSC overexpressing CD::UPRT::GFP exhibited high recovery and prolonged therapeutic gene expression. (**A**) Cryopreserved modified or freshly modified cells were replated onto 6-well plates (*n* = 3). One day later, adherent cells were harvested and enumerated. Percentage recovery was calculated using the number of adherent cells to number of cells seeded into each well. (**B**) Cryopreserved MSC overexpressing CD::UPRT::GFP were replated at 1,000,000 cells/well in the 6-well plate and grown in culture over a period of 5 days. On day 1, 3 and 5 post replating, RFU was measured. On day 5, the cells were harvested from each well and the transfection efficiency was quantified by FACS. Data of biological triplicates (*n* = 3) were presented as mean ± SD. Representative image from each condition is presented

We next assessed the recovery and expression of *MSC overexpressing CD::UPRT::GFP* after cryopreservation and thawing. We determined the recovery efficiency of modified MSCs by assessing the absolute number of adherent cells a day post plating in the cell culture vessel. Evidently, cryopreserved MSC overexpressing CD::UPRT::GFP exhibited a high recovery rate of 88.5% (± 6.35), comparable to the freshly modified MSCs (81.7% ± 3.78) (Fig. 3A).

Next, we determined the sustainability of transgene expression post-thaw. As the intended 5FC dosing cycle was 4 days, we showed that the transgene expression in frozen modified MSCs was sustained with consistent RFU reading of the GFP reporter of up to 5 days post-replating. The transfection efficiency of the cell population decreased from 82.2% (± 0.25) (Fig. 1B, 5 months cryopreserved) to 67.07% (± 0.57) when these cells were grown over 5 days (Fig. 3B). This was likely to be due to the proliferation of the low number of unmodified cells within the population contributing to the apparent lowering of the number of cells transfected. The replating assays for recovery and expression duration revealed that both freshly modified and thawed MSC overexpressing CD::UPRT::GFP retained the typical MSC morphology.

### MSC overexpressing CD::UPRT::GFP retained tumour tropism post-thaw

Tumour tropism of MSCs is well established and involved the SDF-1/CXCR4 axis [34, 35]. While studies have shown that cryopreservation does not affect tumour trophic ability of MSC, these cells were virally modified [18, 36]. In view of the significantly higher payload in the MSCs modified using our method, it was necessary to examine the tumour migratory properties of MSCs expressing high level of transgene post cryopreservation.

**Fig. 4:**
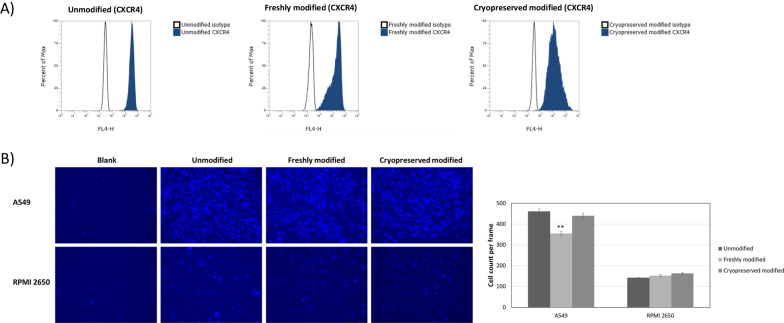
Cryopreservation did not adversely affect the migratory potential of MSC overexpressing CD::UPRT::GFP. (**A**) Unmodified MSCs, freshly modified or thawed MSC overexpressing CD::UPRT::GFP (500,000 cells each group) were stained with anti-human CXCR4 antibody conjugated to a PE fluorophore (Cat. 306,506, Biolegend) at 1:10 dilution. Staining of cells were analyzed by flow cytometry. Percentage of expression was measured using FACS. The population of CXCR4 positive MSCs was gated relative to their isotype controls. (**B**) Four hundred thousand A549 or RPMI 2650 cells were seeded on the bottom chamber of a trans-well (Corning) plate in DMEM supplemented with 10% FBS. Twenty-four hours later, the media was replaced with serum free DMEM before the adding of 150,000 MSC overexpressing CD::UPRT::GFP (unmodified, freshly modified or cryopreserved modified) in the 8 µM matrigel coated cell inserts. The inserts were transferred into the wells containing the cancer cells. Twenty-four hours later, cells on the flip side of the inserts were stained with Hoechst 33,342 and imaged using a microscope. A total of three frames were imaged and counted. The graph represents mean ± SD of migratory cells per frame (*n* = 3). MSC overexpressing CD::UPRT::GFP without cancer cells (blank) served as negative control. Significant differences in cell count per frame between unmodified and modified MSCs were calculated using two-tailed Student’s t test. **P < 0.005

We first examined the expression of CXCR4 using anti-CXCR4 conjugated to PE. We found that both modification and cryopreservation did not significantly affect CXCR4 expression in the canine MSCs. A similar profile (> 90% expression ± 0.02) of CXCR4 was observed in modified and unmodified cells (Fig. 4A). Next, using the matrigel invasion assay, comparable numbers of unmodified, freshly modified or cryopreserved MSC overexpressing CD::UPRT::GFP migrated towards A549 and RPMI 2650 cancer cell-lines (Fig. 4B). Collectively, these data suggest cryopreservation did not adversely affect the migratory potential of canine MSC towards cancer cells.

### Cryopreserved MSC overexpressing CD::UPRT::GFP demonstrated high in-vitro anti-cancer potency

**Fig. 5:**
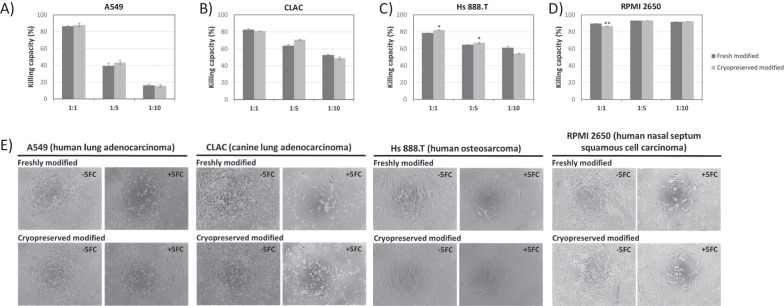
Cryopreserved MSC overexpressing CD::UPRT::GFP exerted strong anti-cancer effect against multiple cancer cell lines. Freshly modified or cryopreserved modified MSCs were co-cultured separately with (**A**) A549, (**B**) CLAC, (**C**) Hs 888.T and (**D**) RPMI 2650. MSC overexpressing CD::UPRT::GFP were added at a ratio of 1 MSC to 1, 5 or 10 cancer cells and subsequently, 150 µg/mL 5FC was added to the wells. After six days, a standard MTS assay was used to determine the cell viability and killing. Conditions without 5FC treatment serve as the controls. (**E**) Representative bright field images of the (1 MSC:1 cancer cell) mixed cultures at the end of the experiment prior to MTS assay. The graph represents data from technical quintuplicates (*n* = 5) presented as mean + SD. Significant differences of the killing capacity between freshly modified and cryopreserved modified MSCs were calculated using two-tailed Student’s t test. *P < 0.05, **P < 0.005

To demonstrate the utility of the cryopreserved cells, we performed a co-culture assay on four different cancer cell lines, namely canine lung adenocarcinoma CLAC and human lung adenocarcinoma A549, osteocarcinoma Hs 888.T, as well as nasal carcinoma RPMI 2650 cell line. After six days in culture, we used the standard MTS assay to monitor cell viability. Interestingly, regardless of cancer types and irrespective of freshly modified or frozen modified MSC, at a ratio of 1 MSC to 1 cancer cell, MSC overexpressing CD::UPRT::GFP achieved a killing efficiency of ~ 80% (Fig. 5A–D). Predictably, a dose response was observed where the killing capacity increased with increasing ratios of MSCs to cancer cells. Of note, with three cancer lines (CLAC, Hs 888.T and RPMI 2650), a killing capacity of > 45% was still observed with 10% of MSC overexpressing CD::UPRT::GFP in the co-culture. These results demonstrated the high potency of MSC overexpressing CD::UPRT::GFP and that the potency was retained post storage (Fig. 5).

### Therapeutic effect of thawed MSC overexpressing CD::UPRT::GFP in canine patient with perianal carcinoma

Previously, we demonstrated the effective long-term suppression of the drug-resistant glioma tumour growth in a subcutaneous mice model and there was no observable adverse effects when using human adipose MSCs engineered with CD::UPRT::GFP [11, 12]. This prompted us to extend the study to treat *no-option-left* canine patients using engineered canine MSCs. Dogs, particularly companion animals, are exceptional models for cancer because of their spontaneous nature and similarity in genetic alterations to human cancers [37, 38]. In this report, we would like to highlight two cases that responded well to treatment.

**Fig. 6:**
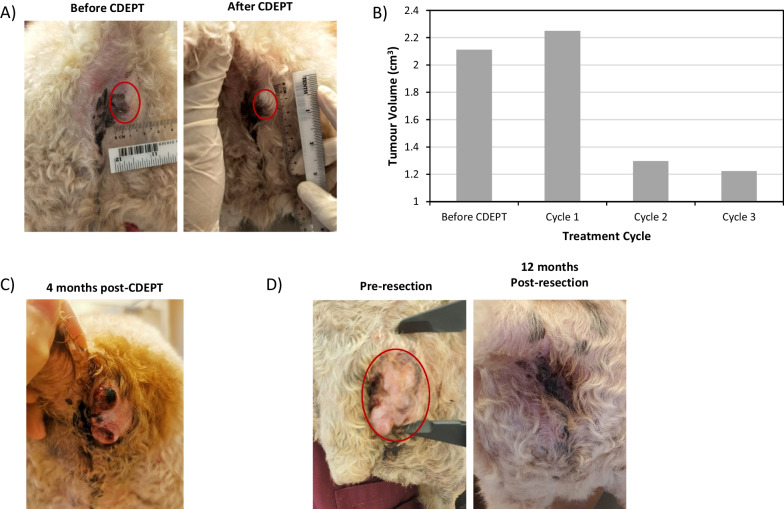
Anti-tumour efficacy of cryopreserved MSC overexpressing CD::UPRT::GFP in a canine patient. (**A**) Reduction in tumour size after GDEPT. (**B**) Tumour volumes measured at the end of each treatment cycle. (**C**) Tumour progression 4 months after completion of GDEPT. (**D**) Images before and 12 months post tumour resection and treatment with GDEPT

A 12-year-old male Poodle presented to a local veterinary clinic with recurring perianal adenoma. Irritation around the area caused difficulty in sitting and defecation. Patient has a history of three recurrences post-surgery, each within 1–2 months post-resection (Table 1). Prior to receiving GDEPT, patient’s SDMA (symmetric dimethylarginine) and creatine were elevated, indicative of possible chronic kidney dysfunction. Apart from this, full blood count and liver function were unremarkable (data not shown). Post treatment, patient blood count remained normal whilst SDMA and creatine continued to be slightly elevated. After the completion of 3 treatment cycles, tumour size significantly decreased (Fig. 6A,B), and a review at 2 weeks and 1-month post treatment indicated that the disease had stabilised. Throughout the course of GDEPT treatment, patient experienced an improved quality of life, significant weight gains and a healthier coat appearance. The tumour also became more compact and circumscribed with less bleeding and inflammation. Unfortunately, 4 months after treatment, a progression in tumour size was observed (Fig. 6C) and a decision was made to surgically remove the residual tumour. Several pre-clinical and clinical studies [39, 40] demonstrated the potential use of modified MSC as adjuvant therapy to eliminate residual cancer cells post-surgery. To further improve the clinical outcome for this patient, after surgical removal of the tumour, 1 × 10^7^ MSC overexpressing CD::UPRT::GFP were injected around the cavity wall followed by a regular cycle of oral 5FC. Remarkably, at time of manuscript preparation, the patient remained disease free for 22 months (Fig. 6D) and continued to experience good quality of life. Table 1 outlines the details of the treatment and patient history.

**Table 1:** Patient history and treatment details (12-year-old, Male, Poodle); series of events in chronological order

Type of intervention	Details	Remarks
*Pre-GDEPT*		
Surgery (lumpectomy)	(1) Lumpectomy of perineal mass; Also (2) had mass on jaw removed	Tumour samples were sent for histopathological analysis and confirmed to be (1) perianal adenoma and (2) acanthomatous ameloblastoma. Perianal mass was completely excised
Surgery (mandibulectomy)	Partial mandibulectomy was done	Routine consultation revealed oral acanthomatous ameloblastoma has grown; scheduled surgical consult with specialist for mandibulectomy
Surgery	Perineal mass recurred; had castration done as well	Castration was done to prevent recurrence

	Route of administration	Dosage of MSC_CD::UPRT::GFP (volume injected)	Oral 5-FC taken daily for 4 days (mg)	Patient weight (kg)	Quality of life observation
*GDEPT treatment*					
GDEPT (cycle 1)	Intra-tumoral	1 × 10^7^ cells (500 µL)	500	8.95	Tumour remained stable. No vomiting and diarrhea. Patient remained active and had good appetite
GDEPT (cycle 2)	Intra-tumoral	1 × 10^7^ cells (500 µL)	500	9.2	Significant weight gain and increased fur growth. Less irritable. Significant decrease in tumour volume
GDEPT (cycle 3)	Intra-tumoral	1 × 10^7^ cells (500 µL)	500	9.15	Tumour volume remained stable. Improved coat condition and fur growth. Patient remained active
Tumour progression observed 4 months post cycle 3					
Surgical resection followed by GDEPT	Intra-cavity	3 × 10^7^ cells (1000 µL)	500	–	22 months cancer free

The second case to highlight involved intravenous administration of the cells. A 11-year-old Toy Poodle presented with severe and persistent coughing and CT scans revealed a nodule of 1.5 cm diameter present in the left caudal lobe of the lungs. The patient was reported to cough incessantly. Prior to GDEPT the patient had undergone surgery for the removal of a prescapular mass.

**Fig. 7:**
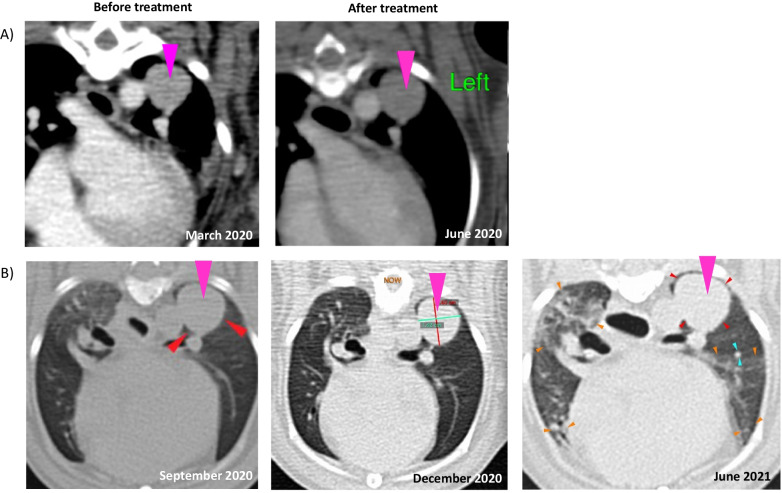
Computed tomography (CT) scan images of the lungs of a 11-year-old male Toy Poodle. Images showing a soft tissue nodule (arrow) of approximately 1.5 cm in diameter in the craniodorsal aspect of the left caudal lung lobe (**A**, left panel). After four treatment cycles, comprising of 1.5 × 10^7^ cryopreserved MSC overexpressing CD::UPRT::GFP administered intravenously followed by four consecutive days of 500 mg 5-FC, the nodule was found to be stabilized (**A**, right panel). CT scan images from 3 months (**B**, left panel), 6 months (**B**, middle panel) and 12 months (**B**, right panel) post treatment

In a similar treatment regime, this patient received 1.5 × 10^7^ MSC overexpressing CD::UPRT::GFP intravenously followed by daily oral 5FC for four consecutive days, resulting in the stabilization of the disease (Fig. 7A). The patient received a total of five treatment cycles. A year after treatment, follow-up scans revealed a slight increase in the size of the solitary pulmonary nodule measuring ~ 1.5–1.8 cm in diameter (Fig. 7B). Interestingly, patient still had no other clinical symptoms (coughing). The patient continued to experience a good quality of life at the time of manuscript preparation, 426 months post treatment. An account of the patient history and treatment details are highlighted in Table 2.

**Table 2:** Patient history and treatment details (11-year-old, Male, Toy Poodle); series of events in chronological order

Type of intervention	Details	Remarks
*Pre-GDEPT*		
Surgery	Prescapular mass removed	Patient presented to vet with incessant coughing. CT scan of the lungs revealed a 1.5 cm diameter nodule present in the left caudal lobe of the lungs

	Route of administration	Dosage of MSC_CD::UPRT::GFP (Volume injected)	Oral 5-FC taken daily for 4 days (mg)	Patient weight (kg)	Quality of life observation
*GDEPT treatment*					
GDEPT (Cycle 1)	Intravenous	1.5 × 10^7^ cells (7.5 mL)@Infused at 3 mL/hr	500	3.75	Patient experienced less coughing post-treatment
Break in treatment due to COVID-19 lockdown					
GDEPT (Cycle 2)	Intravenous	1.5 × 10^7^ cells (7.5 mL) @Infused at 3 mL/hr	500	3.65	Patient responded well to treatment. No adverse reaction was observed
GDEPT (Cycle 3)	Intravenous	1.5 × 10^7^ cells (7.5 mL) @Infused at 3 mL/hr	500	3.45	Patient continues to respond well
GDEPT (Cycle 4)	Intravenous	1.5 × 10^7^ cells (7.5 mL) @Infused at 3 mL/hr	500	3.70	Less coughing observed
GDEPT (Cycle 5)	Intravenous	1.5 × 10^7^ cells (7.5 mL) @Infused at 3 mL/hr	500	3.85	Post-treatment CT scan revealed stable disease. Lung nodule did not grow in size
*Follow up actions*					
CT scan	Three month follow up				Disease continues to be stable. No progression of nodule size, nor extraordinary symptoms. Patient continues to experience good quality of life
CT scan	One year follow up				Lung nodule of interest slightly increased in size. Incidental small nodule and lymph node enlargement was also reported. Suspected metastasis. All other symptoms remain unremarkable

## Discussion

Previously, we demonstrated the high efficiency non-viral transfected MSCs to substantially carry a higher payload resulting in greater potency in cancer killing as compared to lentiviral transduced cells [12]. To realize the clinical translation of these research findings, it is essential to develop frozen working cell banks that are scalable and comply with stringent quality control testing *prior* to use in patients. The current study extended the investigation into the effect of cryopreservation on the over-expression and function of these therapeutic gene modified MSCs in vitro and in *no-option-left* canine patients. These cells can be successfully cryostored without affecting MSC ISCT criteria [41], cell viability, transgene expression or function as a cancer targeting vehicle.

Despite the apparent established practise of cryostoring unmodified MSCs for clinical use, cryopreservation and preparation of MSCs is still a major concern [42, 43]. Galipeau et al. were among the first that demonstrated thawed MSCs can undergo a substantial heat shock response related to the freeze-thawing process [44–46]. A phase 2 trial was unable to meet the primary objective as a treatment against chronic acute respiratory distress syndrome despite promising results in phase 1 due to the substantial variability in the viability of freshly thawed MSCs [47]. Additionally, significant number of studies have demonstrated freeze-thawed MSCs can exhibit metabolic functional impairment [44, 45, 48, 49] and altered biodistribution in vivo [50]*.*

However, contrasting effects of cryopreservation on functions of MSCs have been reported [46, 51–53]. Some studies have shown that freeze-thawing has no impact on the therapeutic efficacy of MSCs [18, 46, 51]. These discrepancies may be explained by the use of different freezing media, passage number or mode of application such as vehicle for suspension cells [43]. Thus, the impact of cryopreservation on each cell product should be evaluated in accordance with its target product profile. In our case, the intended use of the transiently modified MSCs is cancer treatment. To the best of our knowledge, there has been no report that assess the effects of freeze–thaw on such cell product.

Here, we explored the use of a pre-formulated and cGMP grade CS10, for the ease of future manufacturing process development and regulatory approval. We first look at the minimal release criteria for human MSC trials, according to the ISCT standard [9, 41]. Evidently, the freeze–thaw process did not significantly affect the cell viability (Fig. 1A), in line with previous studies on cryopreservation of unmodified MSCs in CS10 [16, 54]. There is a general agreement in the literature that ISCT markers and differentiation potential are not affected by cryopreservation [13]. The immediate post-thaw data presented here is in line with this consensus (Fig. 2). It is important to note that differentiation potential are important assays for defining the type of cells but not MSC function. Emerging evidence suggests that paracrine effects rather than transdifferentiation contribute to the functional benefits derived from MSCs [55–57]. Often, MSC differentiation assays are employed as qualitative in preclinical assessments [11–13, 18].

The successful development of *‘off-the-shelf’* GDEPT is dependent on cells retaining their therapeutic functions post freeze–thaw. Majority of the studies examined impact of cryopreservation on functionality of MSC such as their immunomodulatory [46, 51], anti-inflammatory properties [17, 58] and regenerative properties [59, 60]. In contrast to the defined pharmacokinetics of single agent, unmodified MSC therapeutics demonstrate conditional, complex and multifactorial paracrine effect in vitro and in vivo [55–57]. The lack of specific potency assay results in difficulty in assessing impact of cryopreservation on MSC therapy. As we engineered MSC to deliver a suicide gene, we were able to design the study based on the transgene expression and its effectiveness in inducing cellular apoptosis via a defined co-culture assay.

CD::UPRT::GFP expression immediately post-thaw was maintained at > 70% of the total population (Fig. 1A), satisfying the release criteria with reference to the human clinical trial [9, 39]. Next, we examined the tumour tropic property of MSCs post cryopreservation in a matrigel invasion assay [61, 62]. The tumour migratory efficiency and percentage of CXCR4 population did not change when stored in CS10 (Fig. 4). It is satisfying to note that there is no noticeable difference in the anti-cancer potencies of freshly modified and freeze-thawed MSC overexpressing CD::UPRT::GFP against the 4 cancer cell lines, strongly supporting the use of cryopreserved transfected MSC for *off-the-shelf* cell therapy (Fig. 5). Yuan et al. have reported similar observations in cryopreserved virally modified MSCs expressing TRAIL in cancer killing [18]. Nonetheless, it is worthy to note that our product is transiently modified for high payload of intracellular therapeutic protein. This study provides an insight into the feasibility of cryopreserving a transiently modified MSC product.

We extended our study to assess the therapeutic function of MSC in canine patients with recurring cancers. These patients were inflicted with spontaneously occurring cancers with highly comparable pathophysiology to human cancers and notable is that each dog year is estimated to correspond to approximately 2–5 years of human lifespan [38, 63, 64]. After treatment, it was gratifying to note that tumour regrowth was not observed for at least 22 months (Figs. 6, 7). Intriguingly, this unexpected and prolonged suppression of recurrence may be due to an anti-tumour immune response to 5FU, which may lead to the establishment of immune surveillance [65, 66]. Further analysis of the immunological changes will be required to verify this hypothesis. Taken together, we have successfully demonstrated the strong therapeutic potency of the frozen-thawed MSC overexpressing CD::UPRT::GFP.

A potential limitation of this study is the requirement to process cells to remove DMSO which may lead to significant cell loss, increase cost and complexity in workflow. A solution may be to use GMP grade DMSO free injectable cryopreservatives (e.g., Stem-CellBanker DMSO-free (Amsbio), PRIME-XV Stem FreezIS DMSO-Free, CrySOfree), provided these reagents do not affect cellular properties post-thaw, a study yet to be conducted. Additionally, further assessment of therapeutic efficacy by histopathologic evaluation is not possible with canine patients due to the invasive nature of the sample collection procedures and awaits post-mortem analysis. One option is the use of liquid biopsies for serial treatment monitoring, but this is currently limited by the lack of clinically suitable biomarkers for canine cancers [67, 68]. Another limitation is the availability of canine patients with similar afflictions, and we are in the process of increasing recruitment and to conduct multiple-site investigations. With the favourable results from this study, confidence with this mode of therapy will be appealing to veterinary oncologists.

## Conclusion

This study demonstrated transiently DNA transfected MSCs with significantly higher payload can be successfully cryopreserved with no loss of integrity or function in the cGMP commercially available CS10 reagent. From a regulatory and clinical perspective, our approach improved the overall safety of engineered MSC product as it is virus-free, and cryopreservation allows product standardisation. Furthermore, genetically modified MSCs is highly effective against naturally occurring cancers and offer an option to the current treatment regime.

## Supplementary Information

**Additional file 1. Figure S1.** High payload of CD::UPRT::GFP in DNA but not mRNA transfected MSCs. Canine MSCs were modified with CD::UPRT::GFP at various pDNA or mRNA (Trilink, clean cap technology) amount, respectively. One-day post transfection, the fluorescent images were captured. Representative images are shown (Top Panel). Then, cells were trypsinised, pelleted and resuspended in 1XPBS for flow cytometry analysis. The mean RFU of MSCs modified with DNA or mRNA at various amount were measured by FACS and presented with the bar graph, *n* = 3.

## Data Availability

Data are available within the article or in its Additional file 1.

## Abbreviations

MSCs: Mesenchymal stem cells
GDEPT: Gene directed enzyme prodrug therapy
CD: Cytosine deaminase
UPRT: Uracil phosphoribosyl transferase
GFP: Green fluorescent protein
5FC: 5-Flucytosine
5FU: 5-Fluorouracil
DNA: Deoxyribonucleic acid
CS10: Cryostor10
HTS: Hypothermosol® FRS
FACS: Flow cytometry
RFU: Relative fluorescence units
SDMA: Symmetric dimethylarginine
CT: Computed tomography
cGMP: Current good manufacturing practice
DMSO: Dimethyl sulfoxide
