# Supplementary figures and images for: Cryopreservation does not change the performance and characteristics of allogenic mesenchymal stem cells highly over-expressing a cytoplasmic therapeutic transgene for cancer treatment
Source: Stem Cell Res Ther. 2022 Nov 14;13:519. doi: 10.1186/s13287-022-03198-z (PMC9663191; doi:10.1186/s13287-022-03198-z)

## Slide 1
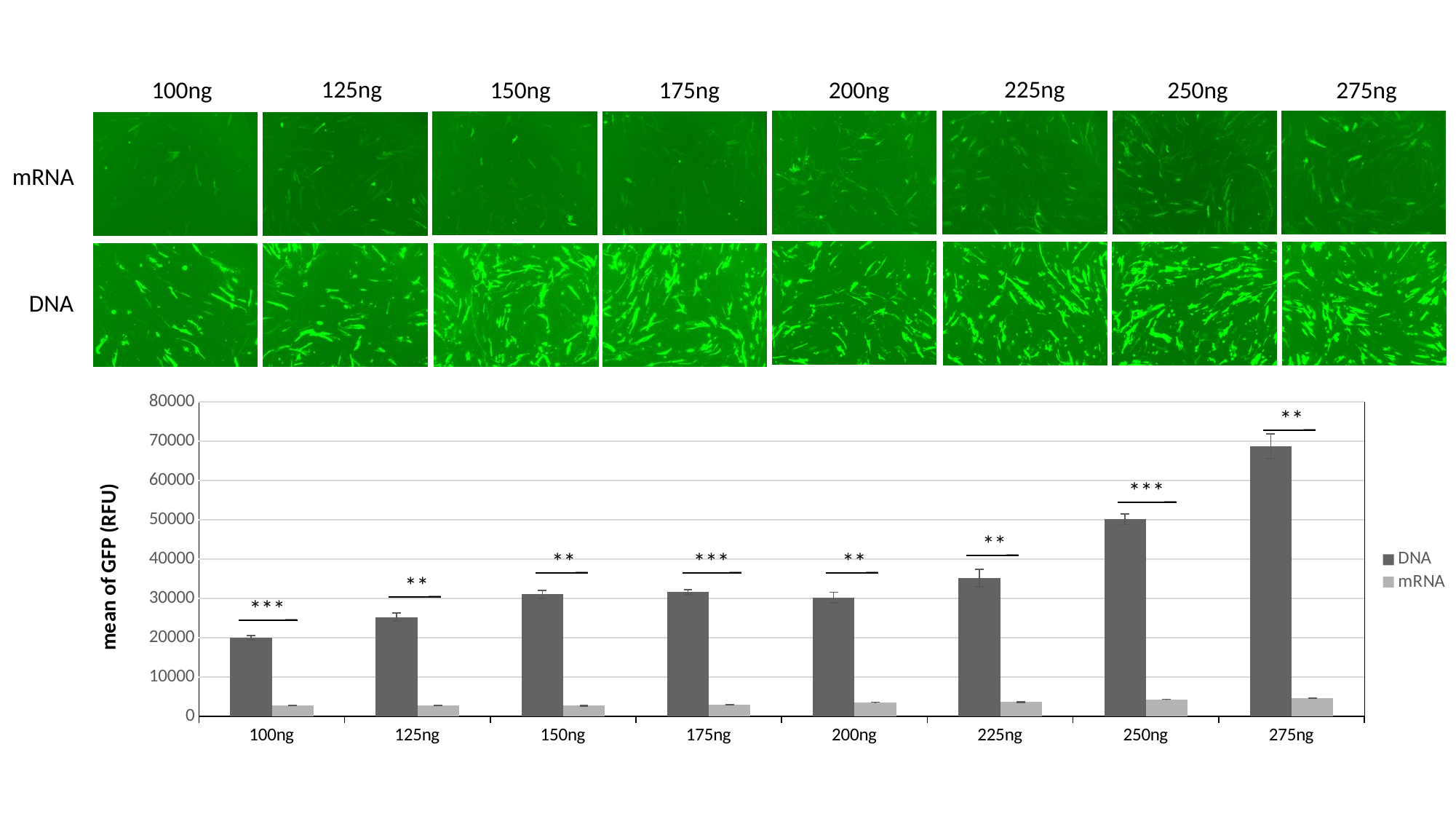

225ng
125ng
200ng
100ng
250ng
150ng
275ng
175ng
### Chart
| Category | DNA | mRNA |
|---|---|---|
| 100ng | 20033.0 | 2904.6666666666665 |
| 125ng | 25296.666666666668 | 2803.3333333333335 |
| 150ng | 31047.333333333332 | 2732.0 |
| 175ng | 31604.0 | 2994.6666666666665 |
| 200ng | 30254.666666666668 | 3636.3333333333335 |
| 225ng | 35206.0 | 3697.3333333333335 |
| 250ng | 50171.0 | 4378.0 |
| 275ng | 68729.66666666667 | 4629.333333333333 | **_
 ***_
 **_
 **_
 ***_
 **_
 **_
 ***_
mRNA
DNA

Supplement: Supplementary file 1 — Additional file 1. Figure S1. High payload of CD::UPRT::GFP in DNA but not mRNA transfected MSCs. Canine MSCs were modified with CD::UPRT::GFP at various pDNA or mRNA (Trilink, clean cap technology) amount, respectively. One-day post transfection, the fluorescent images were captured. Representative images are shown (Top Panel). Then, cells were trypsinised, pelleted and resuspended in 1XPBS for flow cytometry analysis. The mean RFU of MSCs modified with DNA or mRNA at various amount were measured by FACS and presented with the bar graph, n = 3. [file 13287_2022_3198_MOESM1_ESM.pptx]
